# Transdermal Permeation and Anti-Inflammation Activities of Novel Sinomenine Derivatives

**DOI:** 10.3390/molecules21111520

**Published:** 2016-11-17

**Authors:** Zi-Jian Zhao, Chang Zhao, Jing Xiao, Jian-Cheng Wang

**Affiliations:** 1 College of Chemistry and Materials Engineering, Huaihua University, Huaihua 418000, Hunan, China; 2 Huaihua No. 3 High School, Huaihua 418000, Hunan, China; zjzhao72@163.com; 3 College of Chemistry and Chemical Engineering, Hunan University, Changsha 410082, Hunan, China; xiaojing@hnu.edu.cn (J.X.); wjc08071211@163.com (J.-C.W.)

**Keywords:** sinomenine-derivative, transdermal permeation, anti-inflammatory

## Abstract

Sinomenine is extracted from Sinomenii caulis (a traditional Chinese medicine), and it is used as the active ingredient in rheumatic arthritis treatments. It has been used in clinical applications for decades. However, there are some disadvantages, including low activity in transdermal permeation and a high dosage being clinically required. To overcome these defects, sinomenine was used as a primer, and structural modification was performed. In our study, eight new compounds were screened out by transdermal permeation in vitro and anti-inflammatory response in vitro and in vivo. Compound 1a exhibited the most potent transdermal permeation and anti-inflammatory activity. Based on these results, further development of this compound may be warranted.

## 1. Introduction

Rheumatic arthritis (RA) is a chronic autoimmune disease, characterized by cytokine-mediated inflammation of the synovial lining of the joints and destruction of cartilage and bonea. Small joints of the hands and feet are more frequently affected; however, this varies with each individual. RA rapidly progresses to cause joint deterioration and functional disability, eventually leading to unfavorable disease outcomes [1]. RA has significant effects on human health [2]. A worldwide report on the prevalence of arthritis and rheumatism reported the prevalence of RA to be between 1% and 6% [3]. The RA morbidity rate ranges from 1% to 23.1% in certain people [4] (e.g., those who work in the textile or chemical industry or in an underground mine). It is more common between the ages of 35 and 50 years, affecting three times more men than women [1].

Sinomenine (Sin) is an isoquinoline alkaloid that is extracted from the rhizome of Sinomenii caulis (*Sinomenium acutum* (Thunb.) Rehd. et Wils. and *Sinomenium acutum* (Thunb.) Rehd. et Wils.var. *cinereum* Rehd. et Wils.) [5]. Previous studies indicated that Sin was the active ingredient in Rh treatments [6]. However, Sin has some disadvantages, as follows: (1) poor transdermal permeation for external use [7], with a short half-life [8], a large dose required [9]; and (2) releaseing histamine when the cells were stimulated. All these disadvantages results in side effects such as a rash, leukopenia, anaphylactic shock and gastrointestinal reaction [10], when Sin had administered orally or by injection. Although the modified-release preparations of Sin can improve the symptoms [11], it cannot completely eliminate the issues of side effects and a short half-life [12]. Structural modification of Sin can increase the drug efficacy [13]. Synthesis of novel derivatives from Sin to screen for more effective RA treatments drugs has been studied for many years. Some study results have been reported and the drug efficacy has seen great improvement. For example, Wu et al. showed that the Sin derivative linked with the bromine or an aldehyde group at positions 1 and 5 had high anti-inflammatory and analgesia activity [14]. Yao et al. produced several Sin derivatives using structural modification, and found that the derivatives linked by an N or S heterocycle in the carbocycle showed stronger immunosuppression activity than Sin [15]. Wang et al. used Sin as the primer to synthesize several series derivatives, and found that the derivatives having chlorophenyl substituent exhibit much more potent tumor necrosis factor (TNF)-α inhibitory activity than natural Sin and other derivatives [16]. These studies on the structural modification of Sin have placed more emphasis on discovering derivatives with new bioactivity, rather than improving the transdermal permeation of Sin.

**Figure 1:**
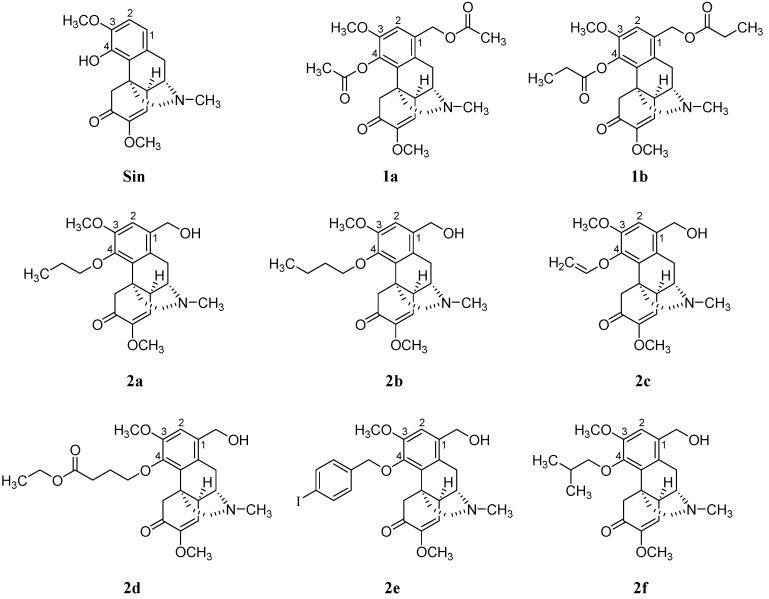
Chemical structures of Sin and its new derivatives (**1a**–**2f**).

In this study, the transdermal permeation and anti-inflammation activities of Sin and its new derivatives (compounds **1a**–**2f**, Figure 1) were evaluated using in vitro transdermal permeation and in vitro inhibition inflammatory interleukin (IL) 1β, IL6 and TNF-α. Moreover, dimethylbenzene-induced mouse ear edema was used to determine whether the enhanced anti-inflammatory effects of some compounds. Other inflammatory animal models were employed to assess the anti-inflammatory effects of compounds in comparison with parent compound. Findings of this study would provide basis in the design of more potent drugs.

## 2. Results

### 2.1. Compound Synthesis

**Figure 2:**
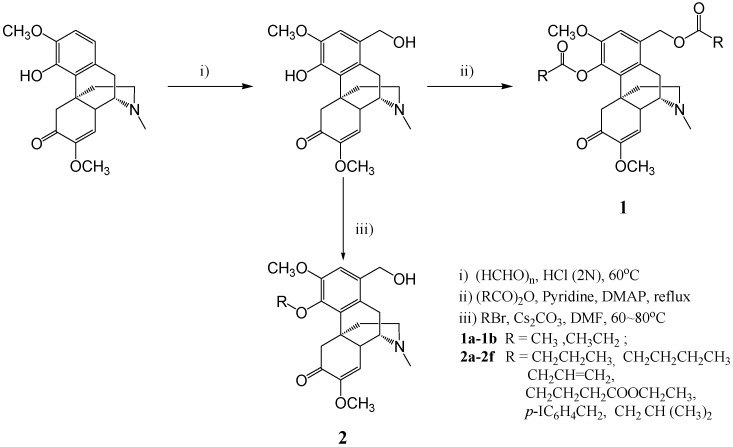
Route of synthesis for sinomenine derivatives.

1-Hydroxymethyl sinomenine was prepared according to Wu’s method [14]. Under the pyridine catalyzation, the intermediate flux reacted with the corresponding anhydride, forming **1a** and **1b** introduced by an ester group at positions 1 and 4. An alkyl was introduced on the phenolic hydroxyl at position 4, forming an ether. In this manner, 6 new compounds **2a**–**2f** were synthesized. Their molecular structures, obtained using ^1^H-NMR ,^13^C-NMR, MS and HRMS, and their synthesis methods are shown in Figure 2. Compounds are described as follows:

*Sinomenine benzyl alcohol intermediate*: a pink solid (62.1% yield), with a melting point (m.p.) of 225–226 °C; ^1^H-NMR) spectrum (CDCl_3_): 1.88–2.04 (m, 3H), 2.43 (m, 3H), 2.45 (d, *J* = 15.6 Hz, 1H), 2.54–2.62 (m, 2H), 3.02–3.09 (m, 2H), 3.25 (t, *J* = 4.00 Hz, 1H), 3.49 (s, 3H), 3.84 (s, 3H), 4.37 (d, *J* = 15.6 Hz, 1H), 4.61 (s, 2H), 5.47 (s, 1H), 6.03 (s, 1H), 6.77 (s, 1H); ^13^C-NMR spectrum (CDCl_3_): 20.57, 35.56, 40.50, 42.73, 47.18, 49.00, 54.83, 56.01, 63.54, 109.32, 109.76, 114.66, 122.78, 127.95, 144.20, 144.70, 152.29, 154.44; and MS (ESI) *m*/*z* (%): 719.0 (2M^+^ + 1, 100).; HREIMS *m*/*z* 359.1743 (calcd. for C_20_H_25_NO_5_ 359.1738).

*1-Acetoxymethyl-4-acetoxy-7,8-didehydro-3,7-dimethoxy-17-methyl-morphinan-6-one* (**1a**): a pale yellow solid (85.2% yield), m.p. 56–58 °C; ^1^H-NMR spectrum (400 MHz , CDCl_3_): 1.86–1.93 (m, 1H), 2.11 (s, 3H), 2.04–2.16 (m, 2H), 2.36 (s, 3H), 2.43 (s, 3H), 2.51–2.55 (m, 2H), 2.66 (dd, *J*_1_ = 6.0 Hz & *J*_2_ = 18.8 Hz, 1H), 3.02 (t, *J* = 7.2 Hz, 2H), 3.27 (t, *J* = 4.0 Hz, 1H), 3.48 (s, 3H), 3.76 (s, 3H), 3.89 (d, *J* = 16.0 Hz, 1H), 5.07 (s, 2H), 5.46 (s, 1H), 6.86 (s, 1H); ^13^C-NMR spectrum (100 MHz, CDCl_3_): 20.78, 20.91, 21.16, 29.66, 36.87, 40.69, 42.63, 45.49, 46.59, 49.95, 54.81, 55.90, 64.10, 112.28, 114.64, 128.59, 130.31, 130.45, 139.47, 149.59, 152.38, 170.73, 192.26; and MS spectrum (EI) *m*/*z* (%): 443.2 (M^+^, 93). HREIMS *m*/*z* 443.1939 (calcd. for C_24_H_29_NO_7_ 443.1927).

*7,8-Didehydro-1-propionyloxymethyl-3,7-dimethoxy-17-methyl-4-propionylox-morphinan-6-one* (**1b**): a pale yellow solid (75.2% yield), m.p. 58–60 °C; ^1^H-NMR spectrum (400 MHz, CDCl_3_): 1.29 (t, *J* = 7.6 Hz, 3H), 1.59–1.62 (m, 1H), 1.71 (t, *J* = 7.6 Hz, 3H), 1.83–1.94 (m, 2H), 2.04–2.09 (m, 1H), 2.38 (q, *J* = 7.6 Hz, 2H), 2.42 (s, 3H), 2.49–2.54 (m, 2H), 2.57–2.73 (m, 3H), 2.99–3.03 (m, 2H), 3.26 (t, *J* = 4.0 Hz, 1H), 3.48 (s, 3H), 3.74 (s, 3H), 3.89 (d, *J* = 16.0 Hz, 1H), 5.08 (d, *J* = 2.0 Hz, 2H), 5.46 (d, *J* = 1.6 Hz, 1H), 6.85 (s, 1H); ^13^C-NMR spectrum (100 MHz, CDCl_3_): 9.14, 20.85, 27.59, 27.89, 36.92, 40.74, 42.68, 45.61, 46.63, 50.09, 54.80, 55.94, 56.01, 64.02, 112.34, 114.76, 128.69, 130.31, 130.53, 139.72, 149.63, 152.50, 174.05, 192.19; and MS (EI) *m*/*z* (%): 471.3 (M^+^, 93). HREIMS *m*/*z* 471.2252 (calcd. for C_2__6_H_33_NO_7_ 471.2249).

*7,8-Didehydro-1-hydroxymethyl-3,7-dimethoxy17-methyl-4-propoxy-morphinan-6-one* (**2a**): colorless oil (80.6% yield), m.p. 97–99 °C; ^1^H-NMR spectrum (400 MHz, CDCl_3_): 1.04 (t, *J* = 7.6 Hz, 3H), 1.83–1.90 (m, 2H), 1.95–1.98 (m, 1H), 2.03–2.15 (m, 3H), 2.42 (bs, 1H), 2.50 (s, 3H), 2.53 (d, *J* = 16.0 Hz, 2H), 2.72–2.80 (m, 2H), 3.07–3.11 (m, 1H), 3.21 (s, 1H), 3.41 (t, *J* = 4.0 Hz, 1H), 3.48 (s, 3H), 3.80 (s, 3H), 3.97–4.10 (m, 2H), 4.21 (d, *J* = 16.0 Hz, 1H), 4.61 (q, *J* = 10.8 Hz, 2H), 5.45 (d, *J* = 1.6 Hz, 1H), 6.87 (s, 1H); ^13^C-NMR spectrum (100 MHz, CDCl_3_): 10.44, 20.96, 23.53, 36.79, 40.81, 42.57, 45.41, 47.20, 49.69, 54.84, 55.79, 56.34, 63.43, 73.80, 111.69, 114.90, 130.18, 132.03, 147.59, 151.30, 152.55, 193.58; and MS (EI) *m*/*z* (%): 401.2 (M^+^, 76). HREIMS *m*/*z* 401.4945 (calcd. for C_2__2_H_21_NO_5_ 401.4942).

*4-(n-Butoxy)-7,8-didehydro-1-hydroxymethyl-3,7-dimethoxy-17-methylmorphinan-6-one* (**2b**): white solid (49.6% yield), m.p. 98–100 °C, ^1^H-NMR spectrum (400 MHz, CDCl_3_): 1.03 (d, *J* = 6.8 Hz, 3H), 1.06 (d, *J* = 6.8 Hz, 3H), 1.8–2.04(m, 7H), 2.15–2.25 (m, 1H), 2.39 (s, 3H), 2.46 (d, *J* = 16.0 Hz, 1H), 2.50–2.56 (m, 1H), 2.61 (dd, *J*_1_ = 6.0 Hz & *J*_2_ = 18.8 Hz, 1H), 2.97 (s, 1H), 2.99 (d, *J* = 17.2 Hz, 1H), 3.18–3.22 (m, 1H), 3.47 (s, 3H), 3.76–3.82 (m, 4H), 3.85–3.90 (m, 1H), 4.20 (d, *J* = 16.0 Hz, 1H), 4.61 (s, 2H), 5.45 (d, *J* = 1.6 Hz, 1H), 6.84 (s, 1H); ^13^C-NMR spectrum (100 MHz, CDCl3): 14.02, 19.17, 20.88, 32.38, 37.01, 40.92, 42.72, 45.70, 47.10, 49.81, 54.76, 56.17, 63.40, 72.00, 111.43, 115.22, 128.06, 130.37, 131.93, 147.59, 151.24, 152.49, 193.68; and MS (EI) *m*/*z* (%): 415.2 (M^+^, 67). HREIMS *m*/*z* 415.2353 (calcd. for C_24_H_33_NO_5_ 415.2345).

*7,8-Didehydro-1-hydroxymethyl-3,7-dimethoxy-17-methyl-4-(2-propen-1-yloxy)-morphinan-6-one* (**2c**): orange solid (75.2% yield), m.p. 58–60 °C; ^1^H-NMR spectrum (400 MHz, CDCl_3_): 1.83–1.91 (m, 3H), 1.93–2.01 (m, 2H), 2.43 (m, 3H), 2.46 (d, *J* = 16.0 Hz, 1H), 2.55 (d, *J* = 11.6 Hz, 1H), 2.65 (dd, *J*_1_ = 5.2 Hz & *J*_2_ = 18.4 Hz, 1H), 3.02 (d, *J* = 18.4 Hz, 1H), 3.05 (s, 1H), 3.27 (brs, 1H), 3.49 (s, 7H), 3.80 (s, 3H), 4.06 (d, *J* = 16.0 Hz, 1H), 4.58–4.68 (m, 2H), 5.01 (d, *J* = 11.2 Hz, 1H), 5.24 (d, *J* = 11.6 Hz, 1H) 5.48 (s, 1H), 6.90 (s, 1H), 7.33 (d, *J* = 8.4 Hz, 2H), 7.72 (d, *J* = 8.0 Hz, 2H); ^13^C-NMR spectrum (100 MHz,CDCl_3_): 20.94, 36.56, 40.95, 42.51, 45.34, 47.08, 49.69, 54.85, 55.65, 56.26, 63.28, 72.47, 93.14, 111.26, 114.92, 127.63, 129.81, 130.26, 132.69, 137.40, 137.92, 146.64, 151.30, 152.56; and MS (ESI) *m*/*z* (%): 386 (M + 1, 100). HREIMS *m*/*z* 385.1169 (calcd. for C_2__2_ H_27_NO_5_ 385.1172).

*4-(n-Butyroxyethyl-1-yloxy)-7,8-didehydro-1-hydroxymethyl-3,7-dimethoxy-17-methyl-morphinan-6-one* (**2d**): yellow solid (85.2% yield), m.p. 56–58 °C; ^1^H-NMR spectrum (400 MHz, CDCl_3_): 1.85–2.05 (m, 3H), 2.41 (s, 3H), 2.48 (d, *J* = 16.0 Hz, 1H), 2.52 (d, *J* = 15.6 Hz, 1H), 2.71–2.77 (m, 2H), 2.95–3.05 (m, 2H), 3.24 (m, 1H), 3.48 (s, 3H), 3.80 (s, 3H), 4.16 (d, *J* = 16.0 Hz, 1H), 4.62 (s, 2H), 4.55–4.73 (m, 2H), 5.27 (dd, *J*_1_ = 10.4 Hz & *J*_2_ = 1.6 Hz, 1H), 5.47 (m, 2H), 6.13–6.23 (m, 1H), 6.86 (s, 1H); ^13^C-NMR spectrum (100 MHz, CDCl_3_): 20.90, 36.72, 40.95, 42.59, 47.08, 45.57,49.61, 54.78, 56.16, 63.23, 72.69, 111.31, 115.15, 117.18, 127.85, 130.31, 132.45, 134.53, 146.83, 151.23, 152.47, 193.85; and MS (ESI) *m*/*z* (%): 474.2 (M + 1, 100). HREIMS *m*/*z* 473.2407 (calcd. for C_2__6_H_35_NO_7_ 473.2402).

*7,8-Didehydro-1-hydroxymethyl-3,7-dimethoxy-4-((4-iodobenzyl)oxy)-17-methyl-morphinan-6-one* (**2e**): pale yellow solid (55.9% yield), m.p. 120–122 °C; ^1^H-NMR spectrum (400 MHz, CDCl_3_): 1.27 (t, *J* = 7.2 Hz, 3H), 1.83–2.00 (m, 7H), 2.10–2.20 (m, 2H), 2.40 (s, 3H), 2.44–2.68 (m, 5H), 2.92–3.04 (m, 2H), 3.21 (brs, 1H), 3.47 (s, 3H), 3.78 (s, 3H), 4.00–4.21 (m, 4H), 4.61 (s, 2H), 5.46 (d, *J* = 2.0 Hz, 1H), 6.85 (s, 1H); ^13^C-NMR spectrum (100 MHz, CDCl3): 0.90, 14.14, 20.78, 25.57, 30.81, 36.81, 40.79, 42.43, 45.36, 46.95, 49.78, 54.70, 55.52, 56.04, 60.26, 62.85, 70.76, 111.20, 115.11, 127.51, 129.91, 132.55, 146.86, 150.98, 152.35, 173.44, 193.59; and MS (ESI) *m*/*z* (%): 474.3 (M + 1, 100). HREIMS *m*/*z* 575.1163 (calcd. for C_27_H_30_NO_5_ I 575.1160).

*4-(i-Butoxy)-7,8-didehydro-1-hydroxymethyl-3,7-dimethoxy-17-methyl-morphinan-6-one* (**2f**): white solid (51.3% yield), m.p. 110–112 °C, ^1^H-NMR spectrum (400 MHz, CDCl_3_): 1.00–1.10 (m, 6H), 1.80–2.00 (m, 7H), 2.16–2.22 (m, 1H), 2.39 (s, 3H), 2.46 (d, *J* = 16.0 Hz, 1H), 2.52 (brs, 1H), 2.60 (dd, *J*_1_ = 6 Hz & *J*_2_ = 18.8 Hz, 1H), 2.97 (d, *J* =1.2 Hz, 1H), 3.00 (s, 1H), 3.19 (brs, 1H), 3.47 (s, 3H), 3.78 (s, 3H), 3.75–3.90 (m, 2H), 4.21 (d, *J* = 16.0 Hz, 1H), 4.61 (s, 2H), 5.46(d, *J* = 1.6 Hz, 1H), 6.84 (s, 1H); ^13^C-NMR spectrum (CDCl_3_): 19.34, 19.39, 20.87, 29.13, 37.02, 40.86, 42.67, 45.64, 47.08, 49.79, 54.78, 55.77, 56.14, 63.34, 78.32, 111.61, 115.22, 128.05, 130.34, 131.97, 147.60, 151.16, 152.49, 193.63; and MS (EI) *m*/*z* (%): 401.4 (M^+^, 55). HREIMS *m*/*z* 401.1190 (calcd. for C_23_H_21_NO_5_I 401.1185).

^1^H-NMR spectra and ^13^C-NMR spectrum for compounds (**1a**–**2f**) can be seen in Figures S1–S16 in Supplementary Materials.

### 2.2. Transdermal Permeation In Vitro

Transdermal permeation of eight Sin derivatives was detected, compared to the Sin. PBS was used to simulate biomedical liquids and HPLC was used to analyze the permeation rate. The results are shown in Table 1. As shown in Table 1, Sin reached its peak (74.87% ± 2.08%) after 8 h. However, the derivatives synthesized in our study showed high transdermal permeation, and the ester derivative was greater than the ether derivative. For example, the permeation rate of **1a** and **1b** reached their peaks within 2 h (98.05% ± 1.79% and 97.28% ± 1.59%, respectively), while it took 6 h for **2a**–**2e** to reach their peaks (85.78% ± 2.21%, 85.36% ± 2.12%, 77.35% ± 2.48%, 82.05% ± 1.87% and 78.31% ± 1.78%, respectively). Compound **2f** reached its peak within 2 h, but the compound was unstable and it decomposed after penetration (2 h). Decomposition was observed in Sin and other compounds all over 8 h.

### 2.3. MTT Assay for Cell Viability

**Figure 3:**
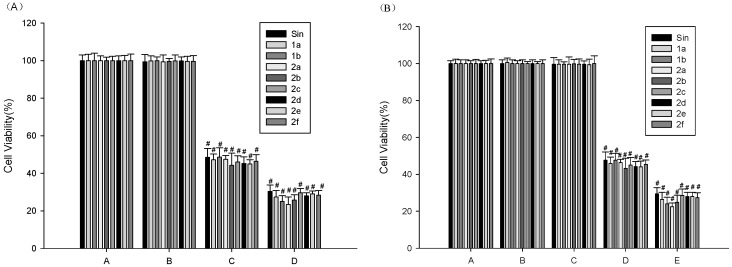
The effects of Sin, Sin derivatives and/or LPS on viability of peritoneal macrophages. (**A**) Cells were treated with Sin and Sin derivatives (final concentrations: A = Vehicle, B = 2 × 10^−2^, C = 5 × 10^−2^, D = 1 × 10^−1^ mg/mL) for 24 h; (**B**) Cells were treated with Sin and Sin derivatives (final concentrations: A = Vehicle, B = Vehicle + LPS, C = 2 × 10^−2^, D = 5 × 10^−2^, E = 1 × 10^−1^ mg/mL and LPS (final concentrations = 10 μg/mL) for 24 h. The results were expressed as mean ± SEM. The results were analyzed by a one-way ANOVA followed by the LSD test (^#^ *p* < 0.001 vs. Vehicle (**A**) or the LPS-treated group (**B**)).

As displayed in Figure 3, the effect of Sin and Sin derivatives on cell viability was determined by 1-(4, 5-dimethylthiazol-2-yl)-3, 5-diphenylformazan (MTT) assay. The results showed that the compounds were cytotoxic to peritoneal macrophages from the concentration of 0.05 mg/mL (*p* < 0.001). Cell viability was not notably affected by up to 0.020 mg/mL of the compounds (>95% cell viability). For this reason, subsequent cell experiments were performed at concentrations less than 0.020 mg/mL, we selected 10 µg/mL as the final concentration of the tested compounds in the subsequent cell trial.

### 2.4. Effect of Compounds on the Levels of IL1β, IL6 and TNF-α in Cell Medium and Lysis of LPS-Stimulated Raw264.7 Cells

**Figure 4:**
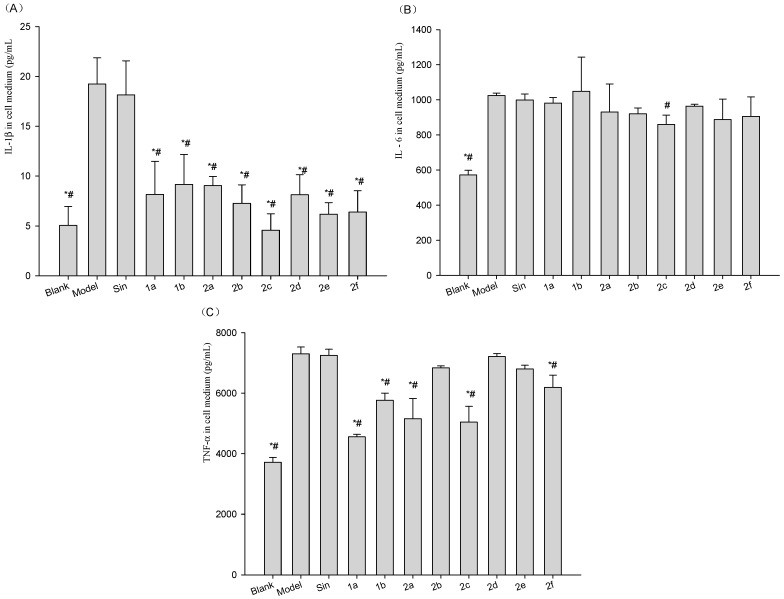
Effect of Sin or **1a**–**2f** on the extracellular secretion of (**A**) IL1β; (**B**) IL6, and (**C**) TNF-α in LPS-treated Raw264.7 cells. Raw264.7 cells were treated with 1 µg/mL of LPS (Blank was untreated), followed by 10 µg/mL Sin or **1a**–**2f** for a total of 12 h. The blank and vehicle (Model) samples were treated with an equivalent volume of arialhicle. Sin did not reduce the LPS-induced increase in IL1β, IL6, or TNF-α, while the **1a**–**2f** derivatives had varying effects on these inflammatory cytokines. Results are expressed as mean ±SD (*n* = 3). * *p* < 0.05 vs. vehicle (Model Group); ^#^ *p* < 0.05 vs. Sin Group.

**Figure 5:**
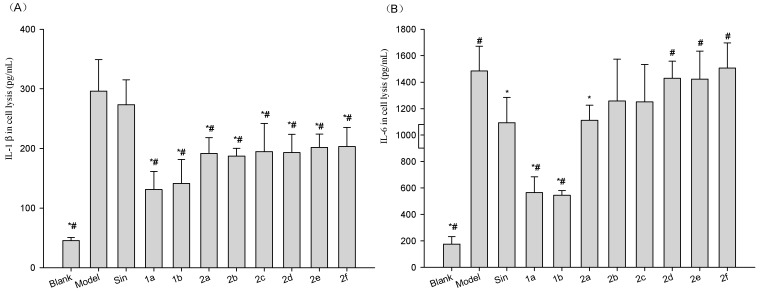

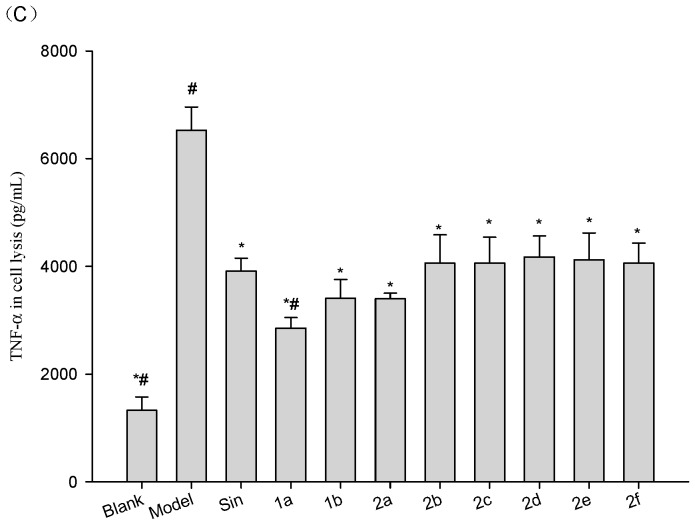
Effect of Sin and **1a**–**2f** on (**A**) IL1β; (**B**) IL6; and (**C**) TNF-α expression in LPS-treated Raw264.7 cells. Raw264.7 cells were treated with 1 µg/mL of LPS (Blank was untreated), followed by 10 µg/mL Sin or **1a**–**2f** for a total of 12 h. The blank and vehicle (Model) samples were treated with an equivalent volume of vehicle. Sin reduced the LPS induced increase in IL6, or TNF-α, while the **1a**–**2f** derivatives significantly reduced all inflammatory cytokines to varying degrees. Results are expressed as mean ± SD (*n* = 3). * *p* < 0.05 vs. vehicle (Model); ^#^ *p* < 0.05 vs. Sin.

To determine if the compounds inhibited the release and expression of the inflammatory factors from Lipopolysaccharide (LPS)-stimulated Raw264.7 cells, we assayed the levels of inflammatory factors in cell medium and lysis from the cells. Results (Figure 4 and Figure 5) showed LPS significantly increased levels of inflammatory factors in cell medium and lysis. Compounds **1a**–**2f** significantly attenuated the level of IL1β in cell medium (Figure 4A). Compound **2c** significantly attenuated the level of IL6 in cell medium (Figure 4B). Compounds **1a**, **1b**, **2a**, **2c** and **2f** significantly attenuated the level of TNF-α in cell medium (Figure 4C).

For inflammatory factor levels in cell lysis, Compounds **1a**–**2f** significantly attenuated the level of IL1β in cell lysis with Sin and model group (Figure 5A), compounds **1a** and **1b** significantly attenuated the level of IL6 in cell lysis with Sin and model group, Compound **2a** significantly attenuated the level of IL6 in cell lysis with model group (Figure 5B). Compound **1a** significantly attenuated the level of TNF-α in cell lysis with Sin and model group, all of the compounds significantly attenuated the level of TNF-α in cell lysis with model group (Figure 5C).

### 2.5. Effect of Compounds on the Expression of mRNA for IL1β, IL6 and TNF-α in LPS-Stimulated Mouse Raw264.7 Cells

**Figure 6:**
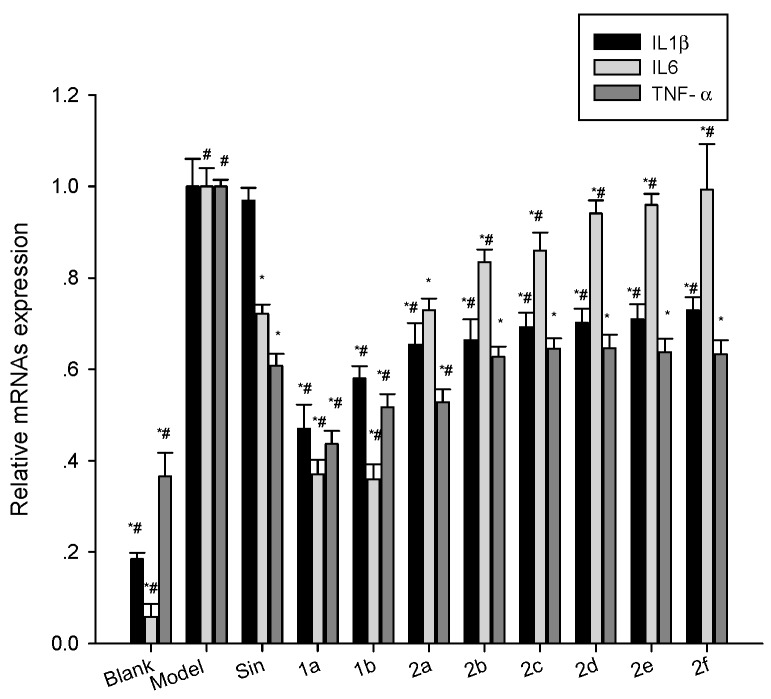
Effect of Sin and **1a**–**2f** on the LPS-induced mRNA expression of the inflammatory factors IL1β, IL6, and TNF-α in Raw264.7 cells. Raw264.7 cells were treated concurrently with 1 µg/mL of LPS (blank was untreated) and 10 µg/mL of Sin or **1a**–**2f** for 12 h. The cells were washed, the mRNA was extracted using Trizol, and the mRNA expression was determined using RT-PCR. In general, the derivatives enhanced the Sin induced IL1β and TNF-α inhibition, while reducing the IL6 inhibition. Results are expressed as mean ±SD (*n* = 3). * *p* < 0.05 vs. vehicle (model group); ^#^ *p* < 0.05 vs. Sin.

To determine if the effect of the compounds were exerted on the transcription of the mRNA for the three cytokines, RT-PCR analysis of the mRNA expression was performed. The effects on the expression of mRNA for IL1β, IL6 and TNF-α in LPS-stimulated mouse Raw264.7 cells are shown in Figure 6. As shown in Figure 6, the anti-inflammatory effects of **1a**, **1b** and **2a** were greater than that of Sin, **1a** and **1b** showed significant inhibition of IL1β, IL6 and TNF-α mRNA expression, **2a** showed significant inhibition of IL1β and TNF-α mRNA expression. Compound **1a** showed the greatest inhibition of IL1β and TNF-α followed by **1b** and **2a**, while **1b** showed the greatest inhibition of IL6, followed by **1a** and **2a**.

### 2.6. Inhibition of Dimethylbenzen-Induced Mouse Ear Edema by Sin Derivatives

**Figure 7:**
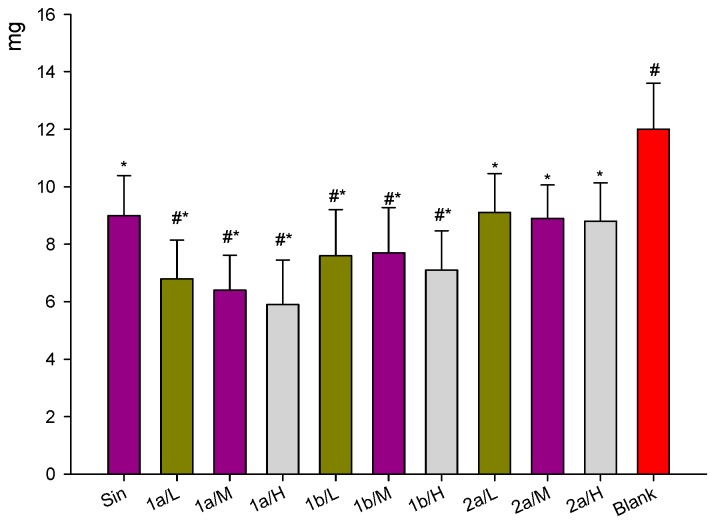
Dimethylbenzene-induced mouse ear oedema was reduced upon treatment with Sin, **1a**, **1b** or **2a** derivatives. Mice were topically treated with Sin or one of its **1a**–**2a** derivatives for 3 days. Ear oedema was then induced by topical administration of dimethylbenzene. Sinomenine and its derivatives significantly reduced the dimethylbenzene -induced mouse ear oedema. Data are expressed as mean ± SD (*n* = 10). * *p* < 0.05 vs. vehicle (blank), ^#^ *p* < 0.05 vs. Sin group. High-dose group: 32.500 mg/kg, middle dose group: 3.250 mg/kg, low dose group: 0.325 mg/kg, Sin group dose: 3.250 mg/kg. Where Sin’s dose (3.250 mg/kg) is equivalent clinical often dose of man.

As shown in Figure 7, Sin (3.250 mg/kg, it is the dosage of people conversion amount in mouse) and its derivatives (**1a**, **1b** and **2a**, 32.500 mg/kg, 3.250 mg/kg, 0.3250 mg/kg) revealed significant (*p* < 0.05) inhibition of ear edema induced by dimethylbenzene in mice compared with the vehicle (blank group). Compounds **1a** and **1b** significantly reduced ear edema at the high, middle, low dose with Sin (3.250mg/kg body weight). Compound **2a** showed more inhibition compared with Sin, but it did not significantly reduce ear edema compared with Sin.

### 2.7. Inhibition of Carrageenan-Induced Rat Paw Edema by Sin Derivatives

To further evaluate the effects of **1a** and **1b** on acute inflammation, we utilized a rat paw edema model induced by carrageenan. As shown in Table 2, the vehicle control group exhibited maximum edema volume around 4 h after carrageenan injection. The derivatives, all significantly (*p* < 0.05) inhibited carrageenan-induced rat paw edema formation compared with the Sin in 6 h. Compounds **1a** and **1b** (at the same dose) were more potent anti-inflammatory agents, and the percentage inhibition of edema was significantly higher than that by Sin throughout the whole course of the experiment, from 1 to 6 h after carrageenan injection. **1a**’s maximum inhibition was 77.24% (32.50 mg/kg body weight; *p* < 0.05) at 1 h. In general, compounds **1a** and **1b** had better inhibition of carrageenan-induced rat paw edema than Sin.

### 2.8. Inhibition of Acetic Acid-Induced Increase in Vascular Permeability

To further evaluate the effects of **1a** and **1b** on edema, Evan’s blue leakage was repeated. Table 3 demonstrates that treatment with Sin or **1a** and **1b** significantly inhibited the acetic acid-induced increase in vascular permeability, compared with the vehicle control. **1a** also significantly inhibited the acetic acid-induced increase in vascular permeability compared with Sin. **1b** did not inhibit the acetic acid-induced increase in vascular permeability more effectively than Sin.

## 3. Discussion

Notably, transdermal administration is an administration route that is worth exploring to help reduce the side effects of administered orally or by injection drug [17]. Because of poor transdermal permeation, there is still no effective Sin external preparation. Previous studies on the structural modification of Sin have placed more emphasis on discovering derivatives with new bioactivity, rather than improving the transdermal absorption performance [13]. To our knowledge, there is no report on increasing transdermal permeation by structural modification of Sin. It is generally known that a substance with high lipophilicity has better permeability [18]. Thus, introduction of an ester or ether at position 1 or 4 of Sin can provide Sin derivatives with higher transdermal absorption performance than the original structure. Thus, we designed and synthesized eight new Sin derivatives, and their permeability and anti-inflammatory effects were studied. In the present study, we observed that the derivatives synthesized in our study showed high transdermal permeation, and the ester derivative was greater than the ether derivative. Similar to literature reports [19], molecular size correlates negatively with diffusivity in skin. For example, **2a** (*n*-butyl) and **2f** (*i*-butyl) have very similar substituents, but **2a** showed greater permeability than **2f**. As shown in the results of transdermal permeation, compounds **1a** and **1b** were the best candidates to be developed into new clinical transdermal anti-rheumatic drugs.

In general, the joint organization of IL1β induced synovitis and cartilage matrix components degradation. IL6 is a key pro-inflammatory cytokine in Rh which was originally described as a T-cell-derived B-cell differentiation factor for the production of antibodies and rheumatoid factor by activated B cells [20]. The previous studies have shown that IL6 plays major role in the pathogenesis of RA [21]. TNF-α is considered one of the crucial pro-inflammatory mediators in joint inflammation and destruction of cartilage and bone of RA patients [22]. Moreover, TNF-α can activate synoviocytes to mediate synovial hyperplasia and produce extracellular matrix-degradative enzymes and chemokines for the development of the arthritic reaction [23]. TNF-α inhibitors such as etanercept, infliximab and adalimumab have been proved to be an effective agent in treating Rh patients not responding to conventional disease-modifying anti-rheumatic drugs [24]. A panel of inflammatory cytokines such as TNF-α, IL1β and IL6 are elevated in the joints of Rh patients for the induction of inflammation, articular destruction, and the co-morbidities [25].

We found that all of the compounds could significantly reduce the release of IL1β, IL6 and TNF-α. Our results also showed that the anti-inflammatory effects of **1a**, **1b** and **2a** were greater than those of the parent compound, as shown by significant inhibition of IL1β, IL6 and TNF-α mRNA expression with the Sin. The result is consistent with the previous finding that Sin ameliorates arthritis via suppressing the production of pro-inflammatory cytokines IL1β and IL6 in collagen-induced arthritic rats [26]. Thus, compounds **1a**, **1b** and **2a** were the best candidates for in vivo anti-inflammation experiments because our results showed that they had better transdermal permeation and anti-inflammatory activity. In vivo anti-inflammation experiments have been performed in the following models: dimethylbenzene-induced swelling of the mouse ear [27], carrageenan-induced paw edema in the rat [28] and Evan’s blue leakage [27]. 

We showed that, at the same dose (3.25 mg/kg), compounds **1a** and **1b** inhibited xylene-induced swelling of the mouse ear more than Sin. Compound **2a** inhibited dimethylbenzene-induced inflammation to a similar degree as that of Sin (*p* > 0.05), but the rate of inhibition was low. Compounds **1a** and **1b** inhibited carrageenan-induced paw edema more than that of Sin and the effect was long-lasting. In addition, compound **1a** significantly inhibited the increase in vascular permeability induced by acetic acid compared with Sin. In summary, our study demonstrated that **1a** is one of the most interesting compounds among the Sin derivatives. **1a** showed stronger transdermal permeation and anti-inflammatory effects than the parent compound and other derivatives.

## 4. Materials and Methods

### 4.1. Materials

#### 4.1.1. Animals

Male Swiss mice weighing 18–22 g and male Sprague Dawley rats weighing 180–220 g were supplied by the Experimental Animal Center of Hunan Province, China. All animals were kept under a 12 h light–dark cycle at a constant temperature (25 ± 3 °C) and relative humidity (60%–70%). Experiments were performed in accordance with the ethical guidelines of the Hunan Province Experimental Animal Management Committee and were in completed in compliance with the National Institutes of Health Guide for the Care and Use of Laboratory Animals.

#### 4.1.2. Drugs and Reagents

Sin was obtained from Shaanxi Sciphar Natural Products Co., Ltd., Xian, China, whereas compounds **1a**–**2f** was synthesized by the College of Chemistry and Chemical Engineering, Hunan University, China. Normal saline was purchased from Sichuan Kelun Group Co., Ltd., Chengdou, China. LPS and MTT were purchased from Sigma Co., Ltd. (St. Louis, MO, USA). Carrageenan was purchased from Tokyo Kasei Kogyo Co., Ltd. (Tokyo, Japan). Dimethylbenzene was obtained from Tianjin Guangcheng Chemical Reagent Co., Ltd. (Tianjin, China). All drugs were prepared with normal saline or DMSO, emulsified with Tween-80 (0.1% *v*/*v*) and dispersed in carboxymethylcellulose (0.5% *v*/*v*). Carrageenan solution (1% *w*/*v*) was prepared with normal saline. RAW264.7 macrophages, IL1β, IL6 and TNF-α kit, purchased from Shanghai Bioleaf Biotech Co., Ltd., (Shanghai, China), and were used for the evaluation of biochemical parameters.

### 4.2. Methods

#### 4.2.1. Compound Synthesis

##### Synthesis of Sinomenine Benzyl Alcohol Intermediate

Sinomenine was dissolved in in HCl (2 mol/L), and mixed with paraformaldehyde at molar ratio 1:2–4 and stirred for reaction at 60 °C for 2 h. The pH of the mixed solution was adjusted to 9–10 using 10% NaOH. The solution was then filtered and washed using dichloromethane. Finally, the sinomenine benzyl alcohol intermediate (1-hydroxymethyl sinomenine) was achieved. 

##### Target Compound 1a and 1b Synthesis

Using pyridine as solvent, the synthesized intermediate (0.5 mmol) flux reacted with acetic anhydride (7.5 mmol) and propionic anhydride (7.5 mmol) catalyzed by dimethylaminopyridine (DMAP, 0.5 mmol) for 3 h. Under reduced pressure, the solvent was removed, and a saturated sodium bicarbonate solution was added to adjust the pH to 8–9. The reaction system was extracted by trichloromethane, washed using water, dried with anhydrous sodium sulfate and filtered. The solvent was evaporated in vacuo, and the residue was subjected to flash column chromatography [V (dichloromethane):V (methanol) = 20–30:1] to give compounds **1a** or **1b**.

##### Target Compound **2a**–**2f** Synthesis

Using DMF as a solvent, the intermediate compound (0.1 mmol) was stirred with bromoalkane (0.11 mmol) at 60 °C for 2 h, and the reaction was catalyzed by Cs_2_CO_3_ (0.2 mmol). The DMF was evaporated in vacuo, and the residue was subjected to flash column chromatography [V (dichloromethane):V (methanol) = 10–15: 1] to give compounds **2a**–**2f**. 

#### 4.2.2. Transdermal Permeation In Vitro

Mice were sacrificed by cervical dislocation and their fur was removed using electrical clippers. The abdominal skin was removed, and the subcutaneous tissue and fat were stripped, washed with saline and stored at 4 °C. An improved Franz diffusion cell method was used with an effective permeability area 2.8 cm^2^ and an accepting cell volume of 6.5 mL [29]. PBS was used as accepting medium, which was degassed using ultrasonic for 30 min before use. The outer layer of skin was tiled upwards and fixed between the diffusion and accepting pool. The accepting cell was filled with PBS (pH 6.8) using a sampling pipe. The liquid level contacted the internal layer of skin, eliminating the bubbles. Sin and its derivative solutions 0.2 mL (10 mg/mL) were accurately measured and placed on the skin surface. After air-drying, the medium was sampled (0.5 mL) after 2, 4, 6, 8 and 10 h at 37 °C with a constant stirring speed of 600 r/min. The solution was filtered using a microfiltration membrane (0.45 μm). An equal volume of fresh medium was added, eliminating the bubbles. The samples were tested using highly effective liquid phase color spectrometer, and the amount of cumulative penetration was calculated, which indicates the transdermal permeation activity of the compound.

All experiments were carried out in quintuple. The percent transdermal permeation activity was calculated using the following formula:

Permeability (%) = Qn/Q_0_ × 100, (1)

where Qn is the sampling point drug permeation quantity; Q_0_ is the initial drug quantity in the donor.

#### 4.2.3. MTT Assay for the Measuring of Cell Viability

Cytotoxic effects of the Sin and its new derivatives on peritoneal macrophages were evaluated by the MTT assay. Cells were cultured in a 96-well plate at a density of 5 × 105 cells/mL. After 24 h, cells were treated with Sin and its new derivatives (final concentrations = 1 × 10^−1^, 5 × 10^−2^, 2 ×10^−2^, 1 × 10^−2^, 1 × 10^−3^, 1 × 10^−4^ mg/mL), LPS (final concentrations = 10 μg/mL), LPS (final concentrations = 10 μg/mL) plus Sin and its new derivatives (final concentrations = 1 × 10^−1^, 5 × 10^−2^, 2 × 10^−2^, 1 × 10^−2^, 1 × 10^−3^, 1 × 10^−4^ mg/mL) for 24 h at 37 °C [30]. After 24 h incubation with or without Sin and its new derivatives, the medium was changed and 20 μL MTT (0.5 mg/mL) was added to all wells. 4 h later the medium was discarded and 150 μL DMSO was added to each well. The plates were agitated gently for 10 min and the optical density at 490 nm was measured by a SpectraMax 250 microplate reader.

All experiments were carried out in quintuple. The percent viability was calculated using the following formula:

Cell viability % = (ODsample − ODnormal)/(ODcontrol − ODnormal) × 100,(2)

#### 4.2.4. In Vitro Inhibition of the Inflammatory Factors, IL1β, IL6 and TNF-α

Mouse Raw264.7 cells were cultured using DMEM with high glucose and 10% FBS with 5 × 10^4^ cells/mL in a 24-well plate. Inflammation was induced by adding LPS (1 µg/mL) into the culture medium. Simultaneously, the cells were treated with Sin and its derivatives (final concentrations = 10 µg/mL) for 12 h. Then, the cell medium was collected for assaying levels of inflammatory factors, e.g., IL1β, IL6 and TNF-α, by using common ELISA kits. Simultaneously, the cells were washed with PBS and also collected for inflammatory factor assays by using the above kits. In another separate trial, we follow same protocol and use RT-PCR method to assay mRNA expressions of inflammatory factors in the cells. The Trizol reagent was used to extract total mRNAs [31]. GAPDH was used for reference gene. SYBR Green I reagent quantitative PCR was used to assay the mRNA expressions of inflammatory factors, e.g., IL1β, IL6 and TNF-α. The primers were synthesized from Invitrogen Company. The examined genes and the primers were shown in Table 4. All samples above were repeated three times. 

#### 4.2.5. Dimethylbenzene-Induced Mouse Ear Edema

Mice were randomly divided into eight groups (*n* = 10), and each group of mice ear was applied topically triple a day for 3 days with vehicle, Sin (standard control), or one of its derivatives (**1a**, **1b**, **2a**) at the tested dosage. Edema was induced on the inner surface of the right mouse ear by topical application of dimethylbenzene (30 μL/ear) 0.5 h after the last drug administration. Forty minutes after xylene application, mice were sacrificed and plugs (8 mm diameter) were removed from both treated (right) and untreated (left) ears at the same location. Edema was measured as weight difference between the plug from the treated (right) ear and the plug from the untreated (left) ear in the same mouse. Anti-inflammatory activity was expressed as weight difference between two ears by comparing mouse ear edema in a treatment group with that in the control group [31].

#### 4.2.6. Carrageenan-Induced Rat Paw Edema

Rats were randomly divided into eight groups (*n* = 10), 0.10 mL of carrageenan (1% in normal saline) was injected to the plantar side of the right hind paw. After 0.5 h, each group of rats right hind paw was applied topically with vehicle, Sin (standard control), or one of its derivatives (**1a**, **1b**, **2a**) at the tested dosage. Edema volumes were measured with a digital plethysmometer (MK-101P, NatureGene Corp., Beijing, China). Volumes of rat right hind paws were measured prior to and at 1, 2, 3, 4, 5 and 6 h after Carrageenan solution or normal saline injection. Anti-inflammatory activity was expressed as edema inhibition ratio. Edema inhibition ratio in various groups was calculated using the following formula [27].

Edema inhibition ratio (%) = (Va − Vp)/Va × 100,
(3)

where Va is mean normal saline group increase of paw volume and Vp is mean experimental group increase of paw volume.

#### 4.2.7. Induction of Increased Capillary Permeability with Acetic acid

After division into groups (*n* = 10), mice received (intraperitoneal) Sin and its new derivatives 3.25 mg/kg (equivalent clinical often dose of man), or vehicle once a day for 5 consecutive days. Two-and-a-half hours after the last treatment, the mice were intravenously injected with 25 mg/kg Evan’s blue solution, and then peritoneally injected with 0.6% acetic acid solution (0.1 mL/10 g body weight). Mice were sacrificed 30 min later, and normal saline (5 mL/mouse) was injected into the abdominal cavity. The abdominal region was gently kneaded, and then the peritoneal fluid was collected from the open abdominal cavity and centrifuged at 3000× *g* for 15 min. Approximately 300 μL of the supernatant was placed in a 96-well microplate. The concentration of Evan’s blue solution was determined on a spectrophotometer using the absorbance at 590 nm. Dye extravasation was quantified using a standard curve [27].

### 4.3. Statistical Analysis

Results were analysed using SPSS 20.0 (SPSS Inc., Chicago, IL, USA). Multiple comparisons were performed using one-way ANOVA followed by LSD t-test. Results were considered statistically significant with a *p* < 0.05. Results are expressed as mean ± SD.

## 5. Conclusions

In this research, eight novel Sin derivatives were prepared as potential transdermal penetration drugs. The ability of these compounds to penetrate mouse skin was tested. All prepared compounds showed higher penetration than Sin. The compounds that exhibited the best penetration in this study were compounds **1a** and **1b**.

The anti-inflammatory activities and penetration study demonstrated that **1a** is the most interesting derivative among the Sin derivatives, with stronger anti-inflammatory effects and penetration than the parent compound and other derivatives. Further studies should determine whether these derivatives can be developed into new clinical anti-rheumatic drugs.

## Figures and Tables

**Table 1:** The permeation rate for Sin and Sin derivative patches through mouse skin.

Compounds	Time (h)				
	2	4	6	8	10
Sinomenine	60.35 ± 2.13	70.03 ± 2.43	72.54 ± 3.15	74.87 ± 2.08	74.48 ± 3.01
**1a**	98.05 ± 1.79 *	98.05 ± 2.01 *	98.05 ± 1.87 *	98.05 ± 1.97 *	93.59 ± 2.53 *
**1b**	97.28 ± 1.59 *	97.28 ± 1.78 *	97.28 ± 2.03 *	97.28 ± 2.05 *	92.46 ± 2.42 *
**2a**	83.05 ± 1.95 *	84.28 ± 3.01 *	85.36 ± 2.12 *	84.57 ± 3.03 *	83.29 ± 2.89 *
**2b**	76.24 ± 2.49 *	76.82 ± 2.23 *	77.35 ± 2.48	76.28 ± 2.55	75.98 ± 2.64
**2c**	78.05 ± 1.79 *	82.05 ± 2.01 *	82.05 ± 1.87 *	81.05 ± 1.97 *	79.59 ± 1.53
**2d**	77.28 ± 2.59 *	77.82 ± 2.03 *	78.31 ± 1.78	77.28 ± 2.05	76.46 ± 2.32
**2e**	79.05 ± 2.13 *	69.25 ± 3.31	62.75 ± 2.51 *	57.81 ± 2.65 *	45.52 ± 2.05 *
**2f**	68.05 ± 2.31 *	69.25 ± 3.31	72.75 ± 2.43	70.81 ± 2.56	68.52 ± 2.55 *

Results are expressed as mean ± SD (*n* = 3 per group). * *p* < 0.05 vs. Sin.

**Table 2:** Anti-inflammatory effect of Sin and its derivatives on carrageenan-induced paw oedema over time in rats.

Compounds	Dose (mg/kg)	Ratio of Paw Volume Increase					
		1 h	2 h	3 h	4 h	5 h	6 h
Sin	3.250	0.1148 ± 0.0157 *	0.1799 ± 0.0209 *	0.1836 ± 0.0158 *	0.1695 ± 0.0126	0.1841 ± 0.0199 *	0.1726 ± 0.0244 *
**1a**	0.325	0.0968 ± 0.0116 *^,#^	0.0711 ± 0.0139 *^,#^	0.1288 ± 0.0236 *^,#^	0.1171 ± 0.0168 *^,#^	0.1334 ± 0.0211 *^,#^	0.1449 ± 0.0251 *^,#^
**1a**	3.250	0.0459 ± 0.0154 *^,#^	0.0710 ± 0.0163 *^,#^	0.0904 ± 0.0157 *^,#^	0.0931 ± 0.0226 *^,#^	0.1129 ± 0.0186 *^,#^	0.1086 ± 0.0181 *^,#^
**1a**	32.500	0.0415 ± 0.0085 *^,#^	0.0583 ± 0.0118 *^,#^	0.0867 ± 0.0154 *^,#^	0.0963 ± 0.0206 *^,#^	0.0945 ± 0.0154 *^,#^	0.1148 ± 0.0277 *^,#^
**1b**	0.325	0.0555 ± 0.0163 *^,#^	0.0803 ± 0.0180 *^,#^	0.1359 ± 0.0273 *^,#^	0.1287 ± 0.0289 *^,#^	0.1432 ± 0.0251 *^,#^	0.1497 ± 0.0219 *^,#^
**1b**	3.250	0.0503 ± 0.0144 *^,#^	0.0728 ± 0.0143 *^,#^	0.1012 ± 0.0197 *^,#^	0.1119 ± 0.0209 *^,#^	0.1250 ± 0.0164 *^,#^	0.1287 ± 0.0262 *^,#^
**1b**	32.500	0.0480 ± 0.0130 *^,#^	0.0702 ± 0.0248 *^,#^	0.0968 ± 0.0242 *^,#^	0.1070 ± 0.0292 *^,#^	0.1098 ± 0.0271 *^,#^	0.1160 ± 0.0314 *^,#^
Model group	Same volume of physiological saline	0.1789 ± 0.0141 ^#^	0.1990 ± 0.0202 ^#^	0.2265 ± 0.0191 ^#^	0.2286 ± 0.0206 ^#^	0.2181 ± 0.0277 ^#^	0.2071 ± 0.0231 ^#^

Results are expressed as mean ± SD (*n* = 10 per group). * *p* < 0.05 vs. vehicle; ^#^ *p* < 0.05 vs. Sin.

**Table 3:** Inhibition of acetic acid-induced increases in vascular permeability.

Group	Dose	OD_590nm_
Vehicle control	/	0.2833 ± 0.0280
Sin	3.250 mg/kg	0.2284 ± 0.0189 *
**1a**	3.250 mg/kg	0.1819 ± 0.0168 *^,#^
**1b**	3.250 mg/kg	0.2279 ± 0.0205 *

Results are expressed as mean ±SD (*n* = 10 per group). * *p* < 0.05 vs. vehicle; ^#^ *p* < 0.05 vs. Sin.

**Table 4:** Details of the genes tested, and the primers used for RT-PCR.

Gene Names	NCBI Accession No.	Primers (5′->3′)	Sizes (bp)
IL-1β	NM_008361	Forward: GAAATGCCACCTTTTGACAGTG	116
		Reverse: TGGATGCTCTCATCAGGACAG	
IL-6	NM_031168	Forward: CTGCAAGAGACTTCCATCCAG	131
		Reverse: AGTGGTATAGACAGGTCTGTTGG	
TNF-α	NM_013693.2	Forward: GGGCTTCCAGAACTCCA	213
		Reverse: GCTACAGGCTTGTCACTCG	
GAPDH	NM_008084	Forward: AGGTCGGTGTGAACGGATTTG	95
		Reverse: GGGGTCGTTGATGGCAACA	

## Supplementary Materials

Supplementary materials can be accessed at: http://www.mdpi.com/1420-3049/21/11/1520/s1.
