# Supplementary material for: Transdermal Permeation and Anti-Inflammation Activities of Novel Sinomenine Derivatives
Source: Molecules. 2016 Nov 17;21(11):1520. doi: 10.3390/molecules21111520 (PMC6273864; doi:10.3390/molecules21111520)
Supplement: Supplementary file 1 [file molecules-21-01520-s001.pdf]

Supplementary Materials: Transdermal Permeation and Anti-Inflammation Activities of Novel Sinomenine Derivatives

Zi-Jian Zhao, Chang Zhao, Jing Xiao and Jian-Cheng Wang

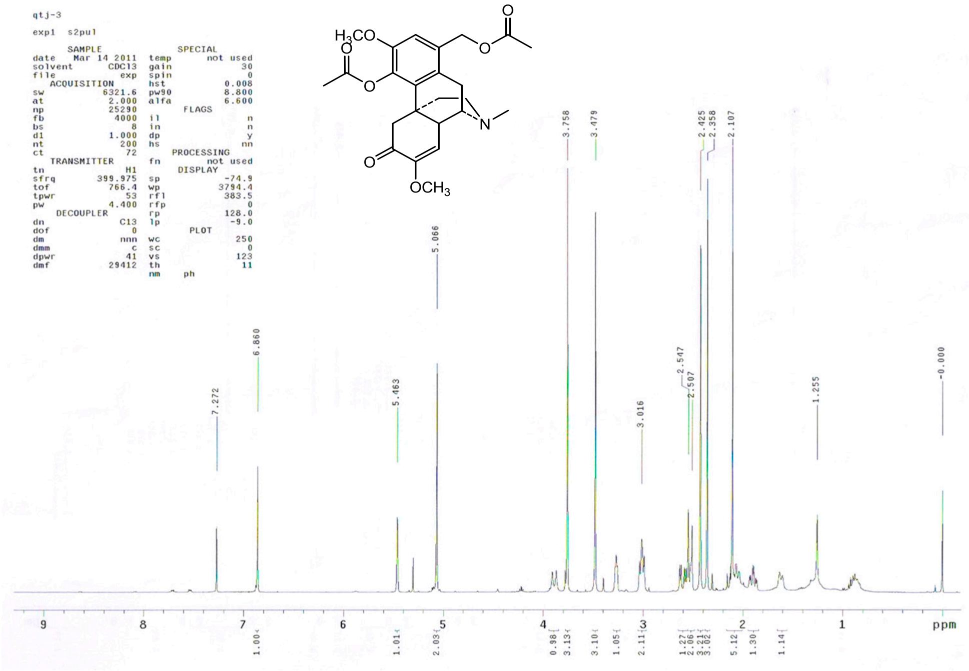

Figure S1. ^{1}H-NMR spectra of compound **1a**.

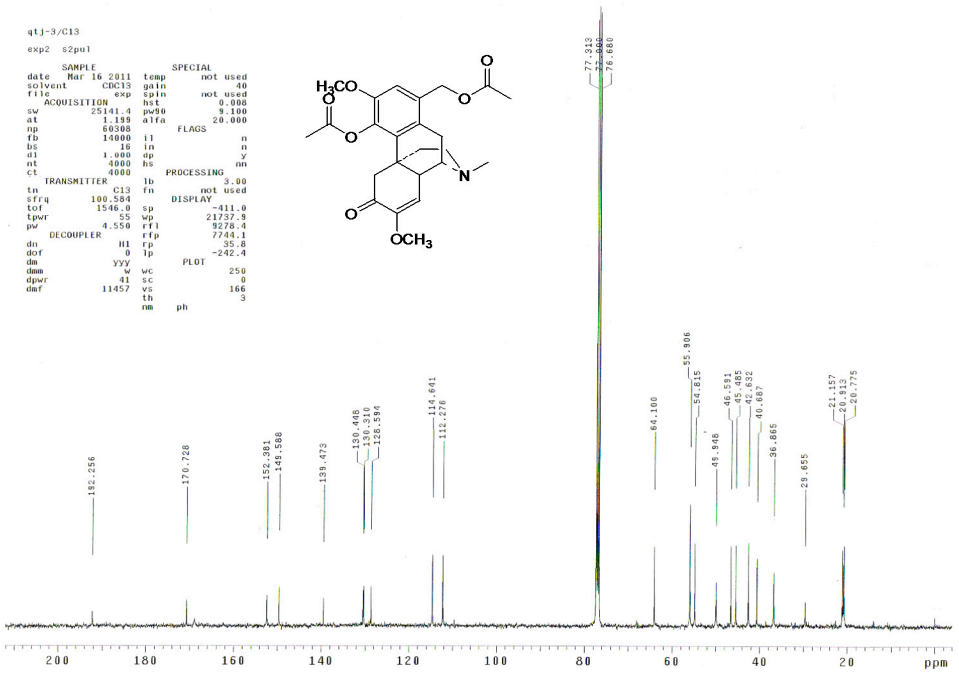

Figure S2. ^{13}C-NMR spectra of compound **1a**.

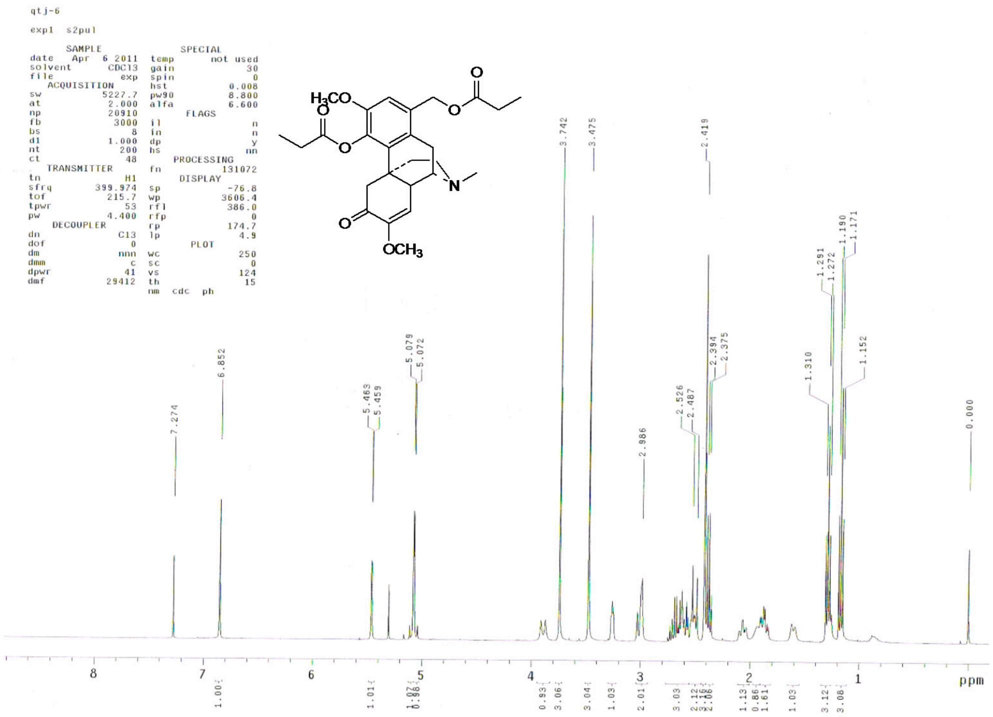

Figure S3. ^{1}H-NMR spectra of compound **1b**.

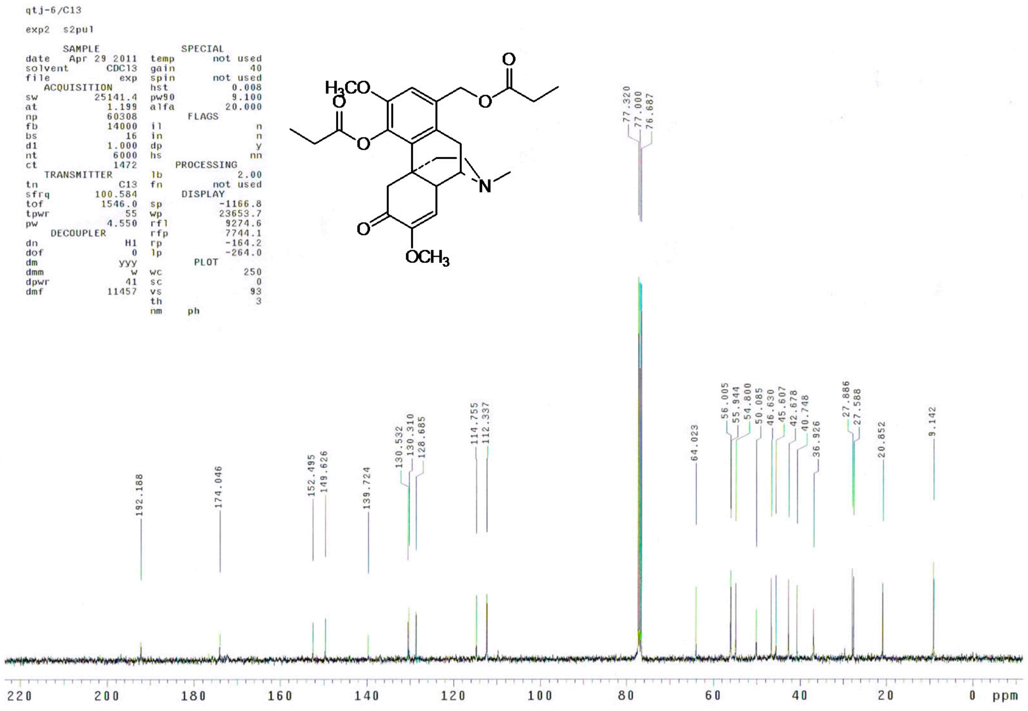

Figure S4. ^{13}C-NMR spectra of compound **1b**.

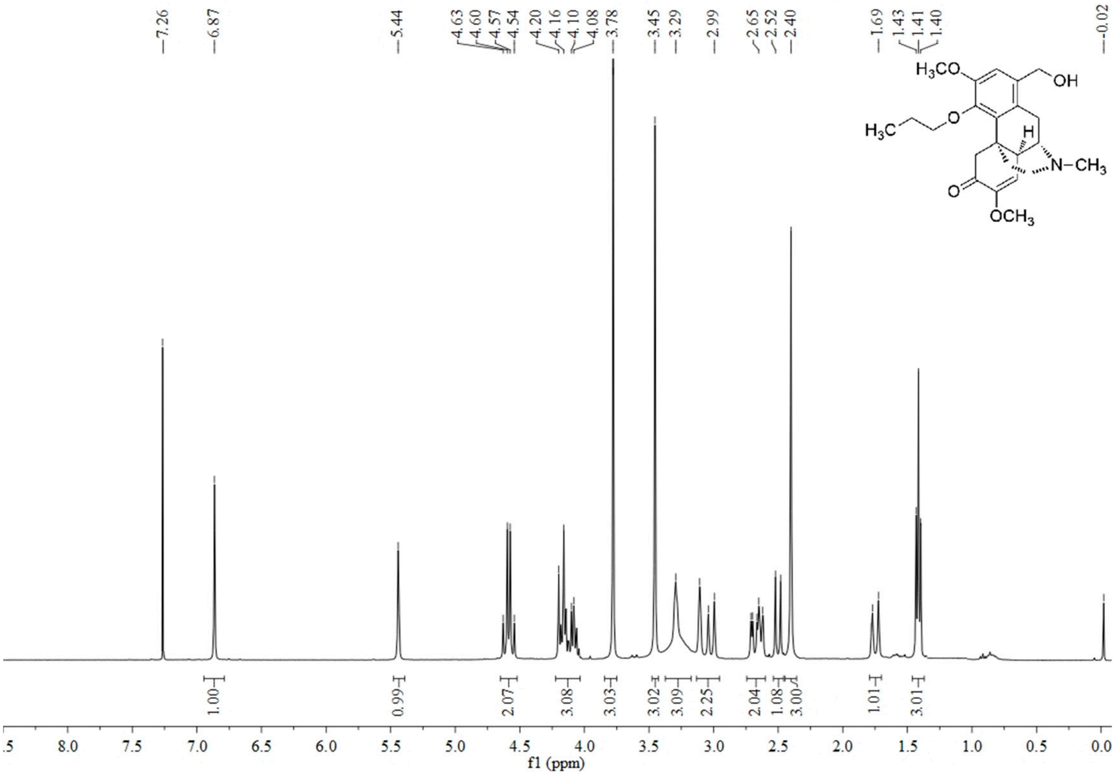

Figure S5. ^{1}H-NMR spectra of compound **2a**.

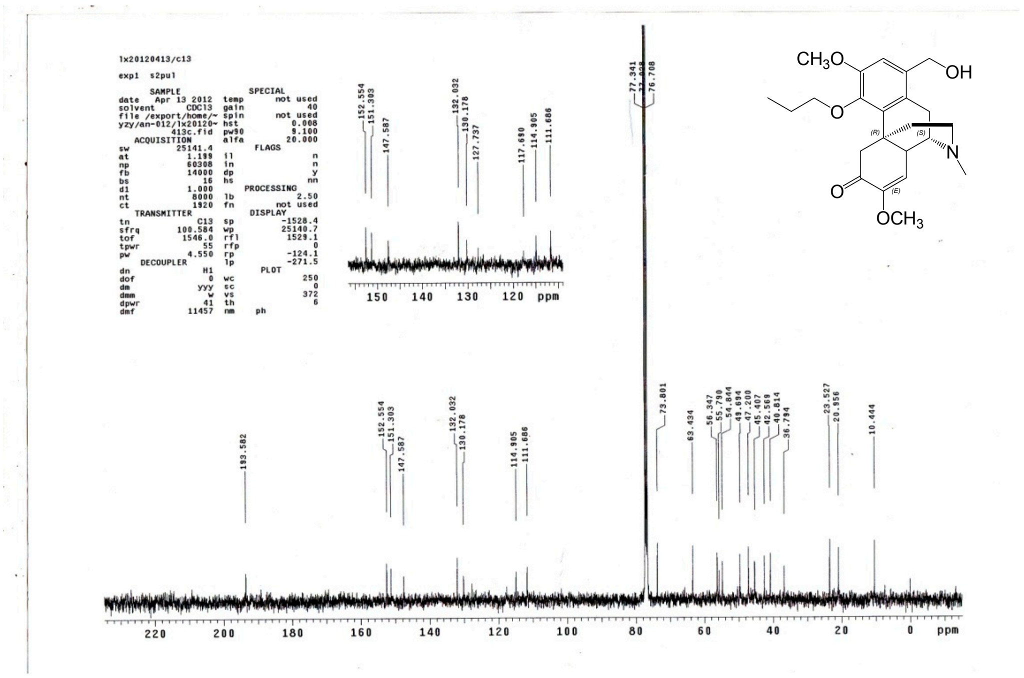

Figure S6. ^{13}C-NMR spectra of compound **2a**.

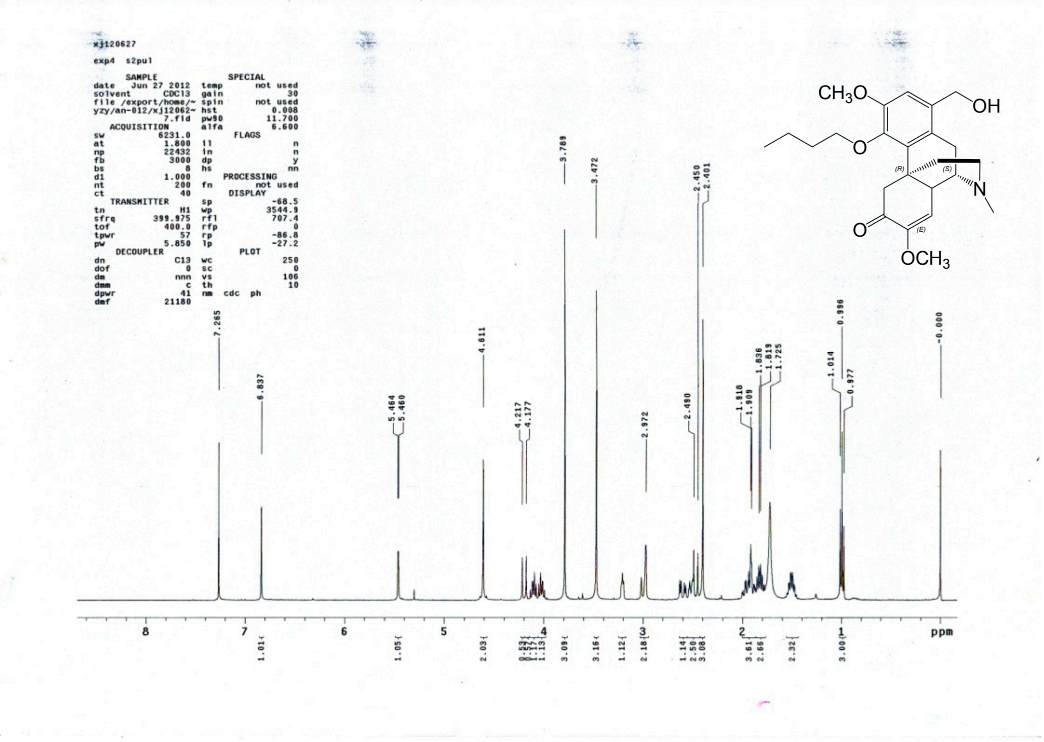

Figure S7. ^{1}H-NMR spectra of compound **2b**.

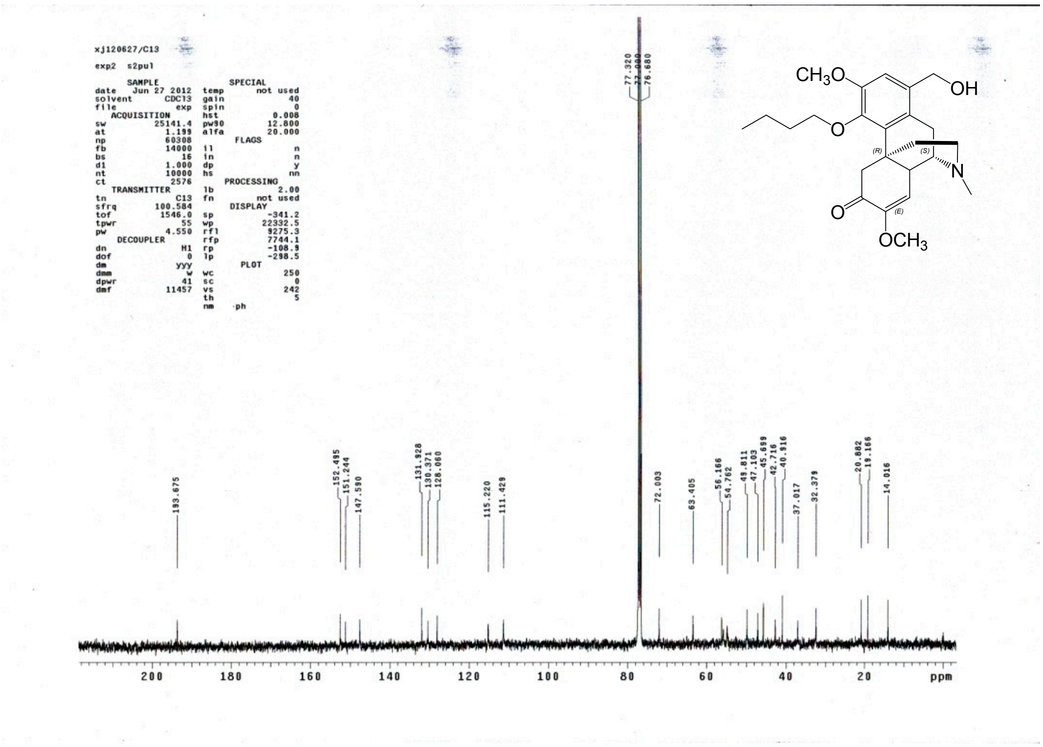

Figure S8. ^{13}C-NMR spectra of compound **2b**.

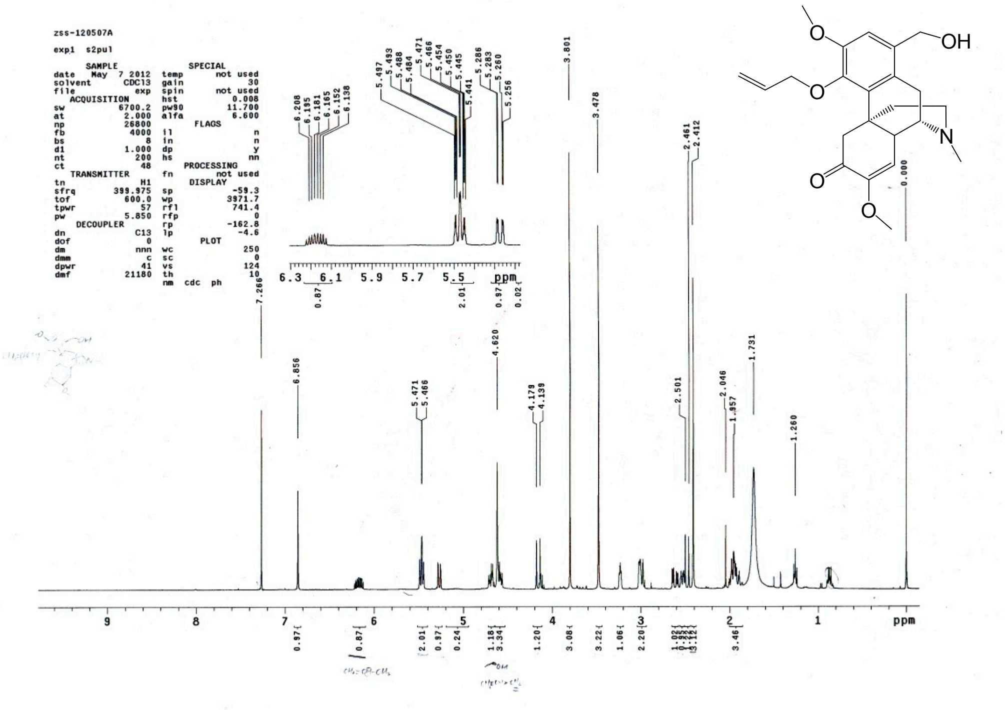

Figure S9. ^{1}H-NMR spectra of compound **2c**.

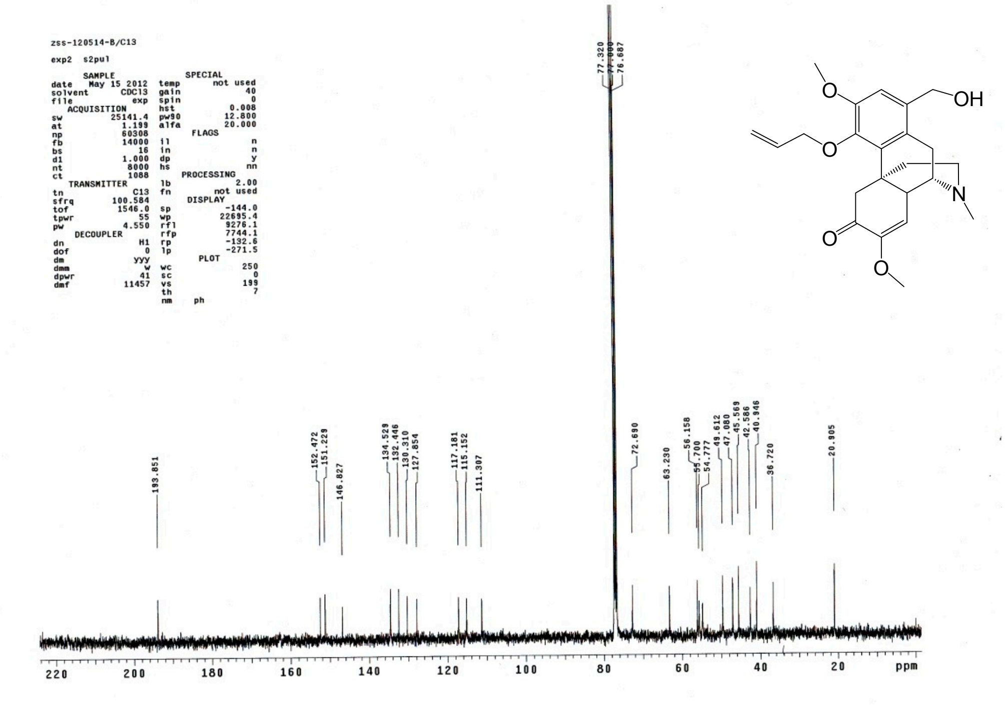

Figure S10. ^{13}C-NMR spectra of compound **2c**.

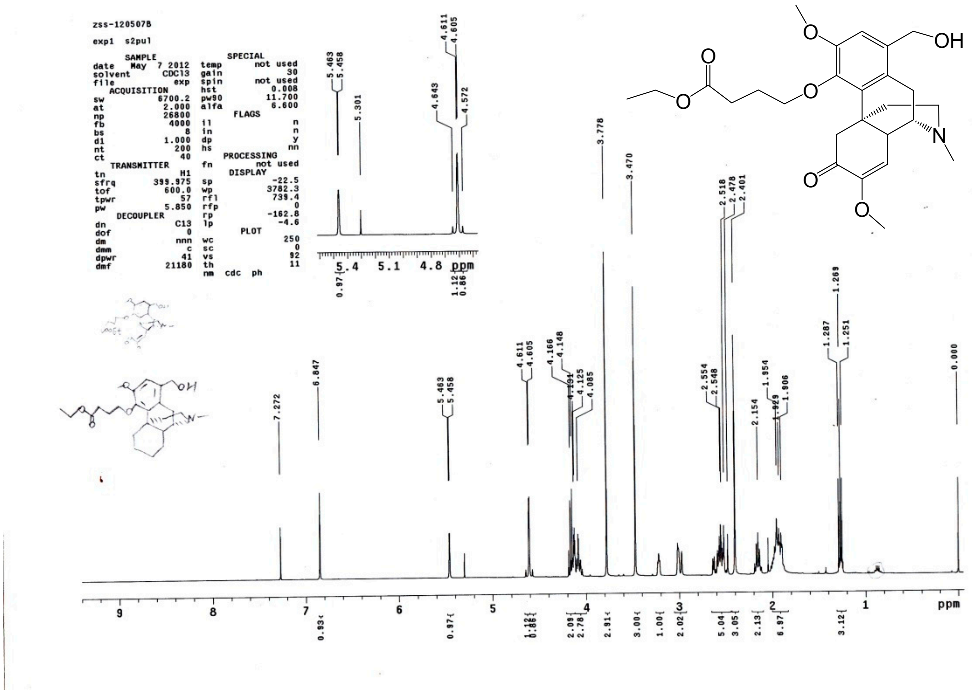

Figure S11. ^{1}H-NMR spectra of compound **2d**.

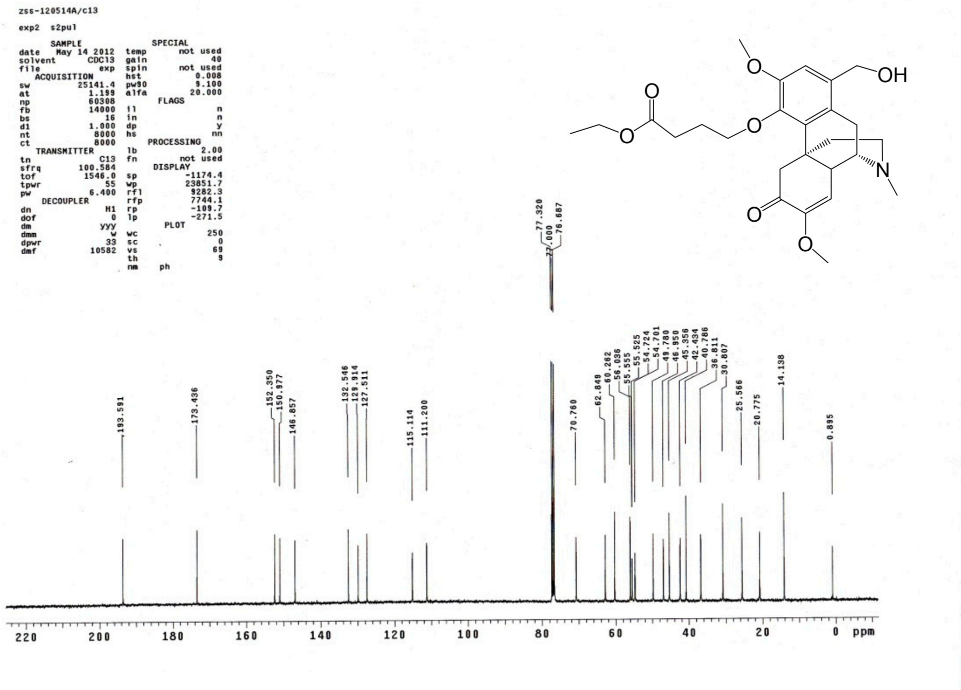

Figure S12. ^{13}C-NMR spectra of compound **2d**.

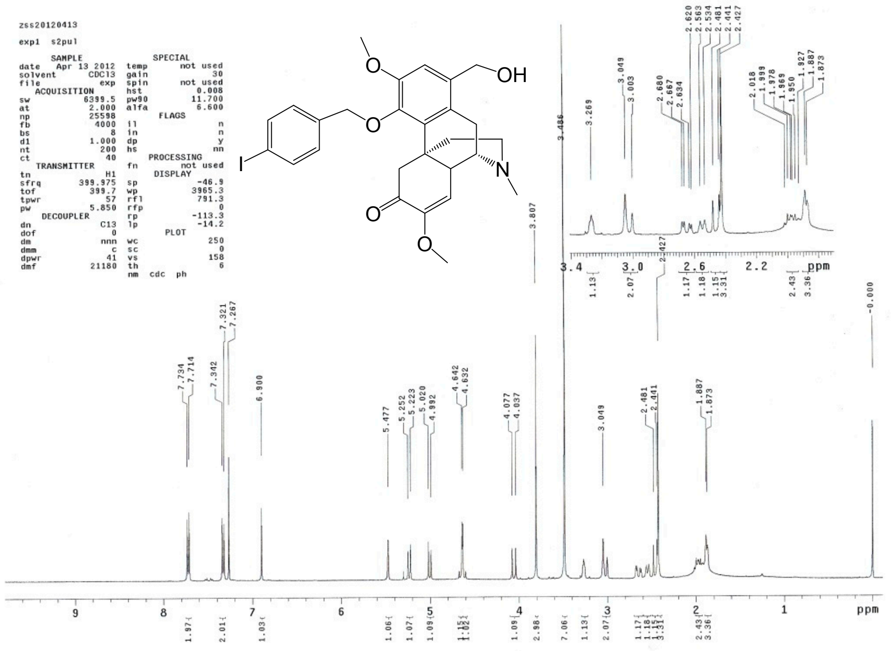

Figure S13. ^{1}H-NMR spectra of compound **2e**.

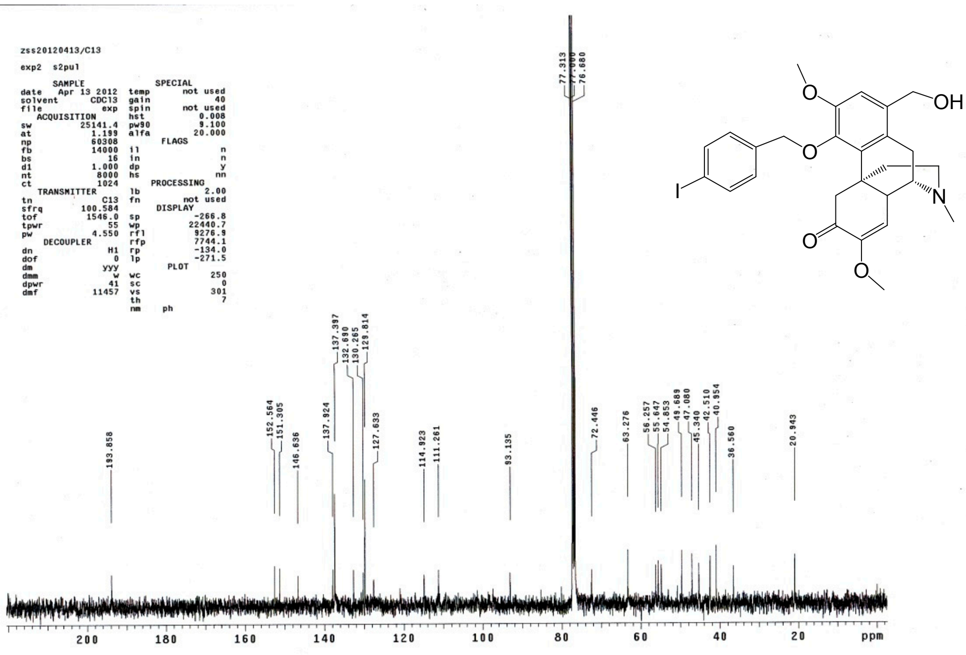

Figure S14. ^{13}C-NMR spectra of compound **2e**.

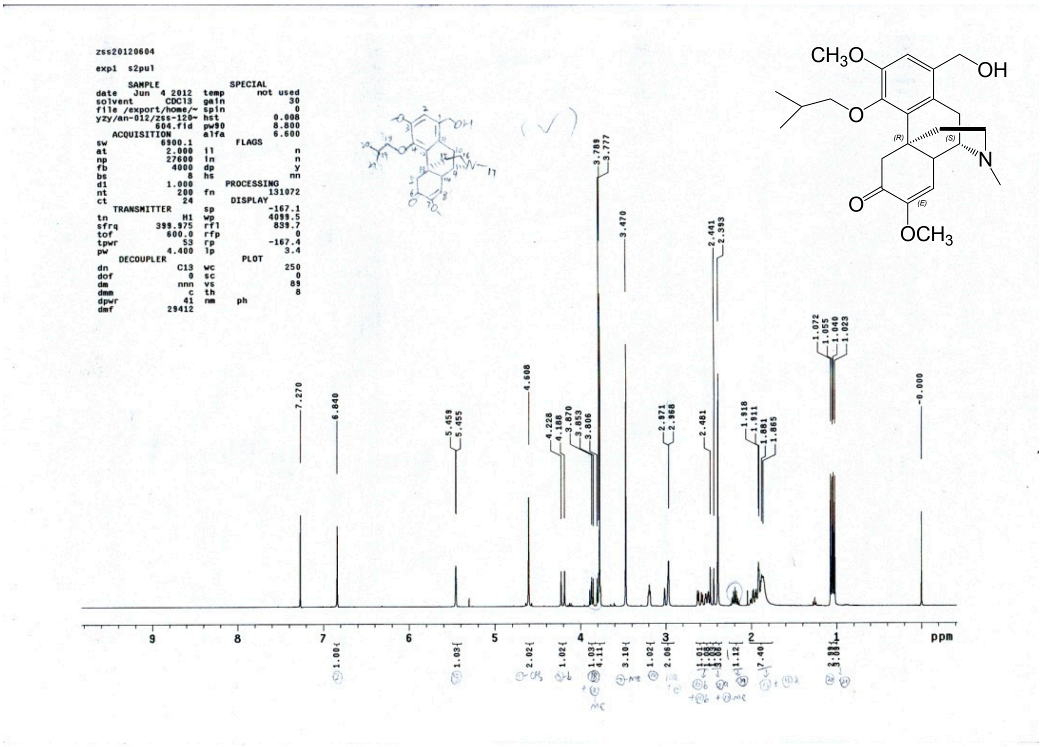

Figure S15. ^{1}H-NMR spectra of compound **2f**.

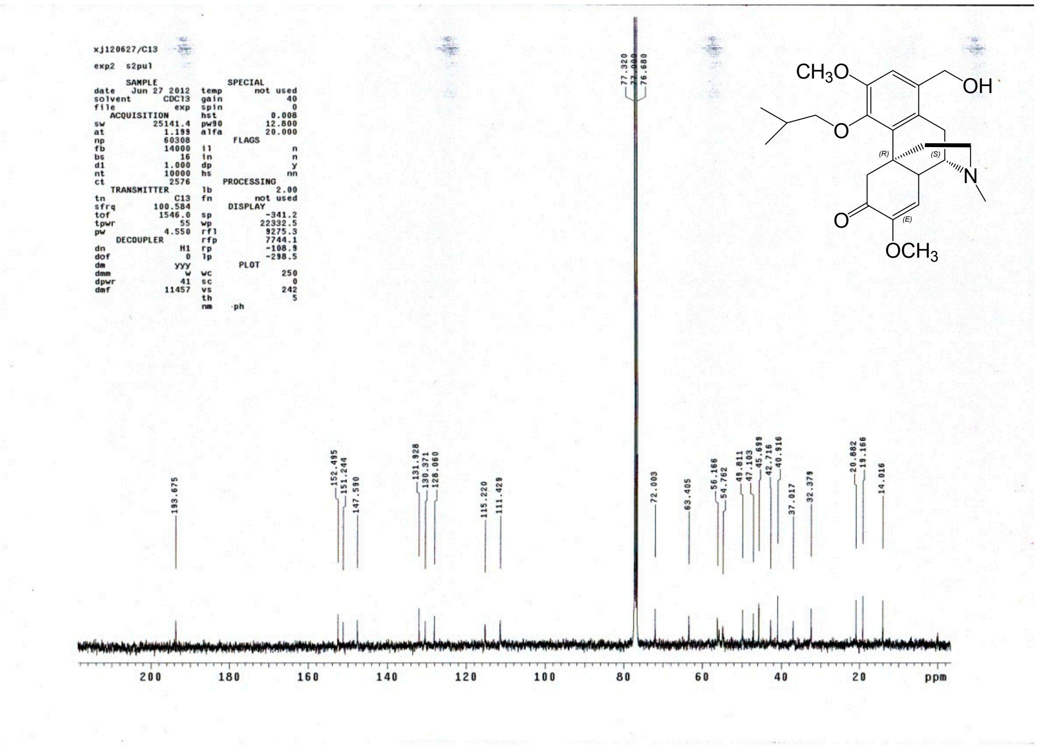

Figure S16. ^{13}C-NMR spectra of compound **2f**.
